# Aging and Repeated Thought Suppression Success

**DOI:** 10.1371/journal.pone.0065009

**Published:** 2013-06-12

**Authors:** Ann E. Lambert, Frederick L. Smyth, Jessica R. Beadel, Bethany A. Teachman

**Affiliations:** Department of Psychology, University of Virginia, Charlottesville, Virginia, United States of America; George Mason University/Krasnow Institute for Advanced Study, United States of America

## Abstract

Intrusive thoughts and attempts to suppress them are common, but while suppression may be effective in the short-term, it can increase thought recurrence in the long-term. Because intentional suppression involves controlled processing, and many aspects of controlled processing decline with age, age differences in thought suppression outcomes may emerge, especially over repeated thought suppression attempts as cognitive resources are expended. Using multilevel modeling, we examined age differences in reactions to thought suppression attempts across four thought suppression sequences in 40 older and 42 younger adults. As expected, age differences were more prevalent during suppression than during free monitoring periods, with younger adults indicating longer, more frequent thought recurrences and greater suppression difficulty. Further, younger adults’ thought suppression outcomes changed over time, while trajectories for older adults’ were relatively stable. Results are discussed in terms of older adults’ reduced thought recurrence, which was potentially afforded by age-related changes in reactive control and distractibility.

## Introduction

From time to time, nearly everyone experiences intrusive, unwanted thoughts [1], [2]. When unwanted thoughts are encountered, individuals may react by intentionally trying to suppress them. While this strategy can be effective at reducing thought recurrence in the short-term, it can lead to increased thought recurrence in the long term (a process called “rebound”), with implications for emotion dysregulation, including development of anxiety and depressive disorders [3]–[6]. Evidence suggests that the eventual rebound in thought recurrence following suppression attempts involves two cognitive processes: a consciously controlled operating process that intentionally tries to suppress occurrences of unwanted thoughts, and an unconscious, uncontrollable monitoring process that scans thought content for suppression failures [5]. Because some aspects of controlled processing decline with age, such as certain inhibition abilities [7], [8], the operating process may be compromised in older adults, leading to age differences in responses to thought suppression attempts that may change over time as controlled processing resources are taxed. However, little research has explored thought suppression outcomes in older adults. To our knowledge, only two published studies to date have used a behavioral thought suppression paradigm with older adults, both conducted by our lab [9], [10]. Surprisingly, the results of these studies generally suggested that degree of thought recurrence does not substantially differ between older and younger adults (Beadel et al. found a trend for older adults to indicate less thought recurrence than younger adults, but it was not statistically significant). However, these studies did not evaluate change in thought recurrence across repeated suppression attempts, examining only total recurrence. Thus, the present study reanalyzes the data from Beadel et al. to provide the first investigation of age differences in thought suppression responses, as resources are likely exhausted across repeated suppression attempts.

Thought suppression paradigms consist of thought suppression periods, where participants are asked to actively prevent a thought from coming to mind and thought monitoring periods, when participants are not instructed to actively prevent the thought from coming to mind, but are asked to simply monitor their thoughts [5]. During both periods, participants are instructed to indicate if the thought does come to mind. Frequency and duration of thought recurrence can be measured based on the number and duration of computer keyboard presses. Our lab’s recent research [9], [10] has found that mean frequency and duration of thought recurrence does not tend to differ substantially between younger and older adults. While Magee and Teachman [9] originally hypothesized that older adults would experience greater thought recurrence than younger adults due to age-related declines in inhibition [11], no age differences in thought recurrence were actually observed. Similarly, Beadel et al. [10] examined age differences in recurrence across four different thought suppression-then-monitor sequences. Again, no significant main effect of age on thought recurrence was observed (using a frequency/duration composite), though younger adults trended toward experiencing greater recurrence. Thus, older adults either show no difference or slightly less recurrence than younger adults, suggesting the role of age-related cognitive changes in thought suppression outcomes is more complicated than originally suspected. In addition to recurrence, we are also interested in subjective ratings of difficulty in reaction to thought suppression and monitoring periods. The way in which subjective reactions to thought suppression attempts change over time may reveal additional clues suggestive of underlying age differences in cognitive processing. For example, Magee and Teachman [11] found that older adults reported more difficulty suppressing thoughts than younger adults during the monitoring period. This finding is consistent with the idea that older adults may compensate for reduced processing resources by more liberally expending what resources they have, resulting in higher perceived difficulty.

One approach to better understand these patterns is to consider how age differences in thought recurrence change over repeated suppression attempts. Empirical evidence suggests that suppression attempts place demands on working memory capacity (see [12]), and the resource-depletion literature strongly suggests that suppressing unwanted thoughts is depleting [13]–[15]. The present paper thus reanalyzes Beadel et al.’s [10] original data to examine responses to multiple suppression-then-monitor sequences, highlighting the impact of repeatedly taxing the controlled processing resources used when trying to suppress occurrences of unwanted thoughts. Whereas Beadel et al. collapsed across suppression attempts (focusing on thought content, regardless of when the thought appeared in the four thought suppression-then-monitor sequences); we employ multilevel modeling to examine age differences in thought suppression reactions over the course of time and repetition. While we examine both suppression and monitoring periods, we predict that age differences will be more likely for suppression (as opposed to monitoring) periods, when the strategically controlled operating process, which is particularly vulnerable to age-related changes, is thought to be most active.

We also consider three accounts of how age differences in thought recurrence and subjective reactions to thought suppression may play out over repeated suppression attempts. Each account is based on a different feature of age-related cognitive change: 1) compensation for older adults’ reduced controlled processing resources (referred to as the Compensation account), 2) older adults’ relative shift from proactive to reactive modes of control (referred to as the Dual-Mechanisms of Control account), and 3) older adults’ openness to self-distraction (referred to as the Distraction account). The accounts are not mutually exclusive, but highlight different mechanisms that might explain age differences in thought recurrence levels and change trajectories over repeated suppression attempts. For most accounts, our predictions for the outcome variables (frequency and duration of recurrence, and suppression difficulty) are similar, because these variables are conceptually related, and are generally expected to covary together; nevertheless, we also note cases where different outcomes might be expected. As such, our consideration of these three accounts in the context of thought suppression is not intended to serve as a theory testing exercise. Rather, our goal is to test for both mean level age differences in reaction to thought suppression and age differences across repeated suppression attempts to refine our understanding of how cognitive aging may affect the experience and consequences of trying to suppress intrusive thoughts.

### Age Differences in Mean Level of Thought Suppression Reactions

Reductions in available processing resources have been used to explain many age-related changes in behavior and cognition [16]–[18], and these changes may impact age differences in mean level of thought suppression efficacy as well. The nature of the impact may vary, however, depending on which age-related theory of cognitive change (e.g., Compensation vs. Dual-Mechanisms of Control) and which thought suppression outcome (e.g., actual thought recurrence vs. subjective suppression difficulty) is considered. For instance, despite considerable evidence of age differences in controlled processing based on neuroimaging findings (e.g., [19]), corresponding behavioral age differences on cognitive task performance are not always observed. This discrepancy is thought to occur in part because of older adults’ ability to compensate for their reduced resources (e.g., based on evidence of over-activation in older adult prefrontal cortices; [20], [21]). If older adults successfully compensate for frontally-mediated deficits in controlled processing by over-recruiting prefrontal regions, they will remain effective at thought suppression, but presumably expend much of their relatively more limited cognitive resources on this compensation effort. This Compensation account would thus predict the absence of age differences in the average level of thought recurrence for both frequency and duration, consistent with Magee and Teachman [9]. However, one would expect older adults to report greater suppression difficulty than younger adults, particularly during suppression periods, which ostensibly demand greater control.

On the other hand, older adults also differ from younger adults in their ability to continuously engage active controlled processing [22], [23]. Specifically, younger adults tend to utilize a proactive control strategy in which task goals are continually maintained in an active state. Conversely, older adults tend to rely more on a reactive control strategy in which task goals are activated on an as-needed basis in reaction to within-task demands. This age-related shift in mode of control, referred to as the Dual-Mechanisms of Control account, could also affect thought suppression outcomes. During suppression, both strategically controlled and automatic processes are thought to operate, but controlled processing may be less fully engaged in older adults given age-related declines in active goal maintenance [23]. As a result, the goal of suppressing unwanted thoughts may be less consistently active. Recall that ongoing suppression attempts are thought to lead to rebound; that is, elevated recurrence of unwanted thoughts in the long run (though not in the short term; [24], [25]). Thus, younger adults may actually experience more recurrence than older adults across repeated suppression attempts given younger adults’ use of more proactively controlled processing during suppression, which can lead to greater subsequent rebound. In other words, this Dual-Mechanisms of control account suggests age differences in overall level of thought recurrence during suppression, such that younger adults experience longer and more frequent thought intrusions than older adults (consistent with the trend observed in [10]). Also, because reactive control does not require continuous engagement of active controlled processing, older adults’ reliance on a reactive control strategy may reduce the overall demand of the task, leading them to report less difficulty in reaction to thought suppression attempts.

Similar predictions follow from evidence suggesting that we become more susceptible to distraction as we age [26], [27]. In some contexts, this distractibility can be beneficial [28], [29]. For instance, Campbell et al. demonstrated that older adults are more likely than younger adults to associate task-relevant information with extraneous information in the environment, a phenomenon referred to as hyper-binding. Within the thought suppression context, because of hyper-binding extraneous cues to the to-be-suppressed thought, older adults may possess more readily available distractors that can aid their thought suppression attempts. Given that self-distraction can be an effective short-term suppression strategy [30], [31], this Distraction account suggests that older adults may be able to use extraneous cues to avoid thinking about the unwanted thought. In this case, younger adults would be expected, on average, to experience more thought recurrence than older adults, and for longer durations. Note, we expect that younger adults will use distraction somewhat also, but they will need to more actively search for distractors, which uses controlled processing resources [31], compared to older adults. Further, if older adults have more readily available, automatically activated distractors, they should perceive suppression to be less difficult than younger adults.

In summary, if older adults are able to successfully compensate for reduced controlled processing resources, mean age level differences may not emerge across the more objective thought recurrence duration and frequency measures. However, compensation may lead older adults to perceive suppression as more difficult than younger adults. In contrast, to the extent that older adults rely on more reactive strategies of control and have more readily available distractions from the unwanted thought, they may show shorter, less frequent recurrence at the mean level than younger adults, and perceive suppression to be less difficult.

### Age Differences in Trajectory across Repeated Suppression Attempts

If older adults compensate for reduced processing resources by more liberally spending what resources they have, then the trajectories of change over time for thought recurrence frequency and duration, and perceived suppression difficulty should differ by age group. Initially, younger adult’s thought recurrences are likely to become shorter and less frequent as the task becomes easier with practice, resulting in less reported difficulty in reaction to thought suppression. However, as resources become depleted across repeated suppression attempts, thought suppression reactions may begin to rise, leading to an uptick of longer and more frequent thought recurrences and higher reports of difficulty on later measurement occasions. Older adults, on the other hand, may experience an immediate need for compensation. If so, the time trajectories for older adults’ reactions to repeated thought suppression attempts would be expected to remain flat (if compensatory efforts can be maintained) or increase (if depletion of resources compromises compensatory efforts over time, or the early compensatory efforts result in greater later rebound).

A similar prediction follows from age differences in use of reactive vs. proactive control, though for different reasons. Over time, for younger adults, the greater cognitive control resources devoted to active suppression via a proactive control strategy may initially lead to a decline in intrusive thought frequency and duration (because suppression tends to initially be effective at reducing recurrence; [24]), but, as these resources are depleted over repeated attempts and proactive control becomes more difficult, frequency and duration may rise in the form of rebound effects. Similarly, younger adults’ reported difficulty should initially decline as they gain practice employing proactive control, only to later rise as they experience rebound brought on by their earlier proactive suppression strategy. In contrast, because reactive control does not require continuous engagement of active controlled processing, older adults’ reliance on a reactive control strategy may lead to relatively flat trajectories across suppression outcomes because the reduced difficulty associated with their reactive control strategy should be more sustainable, and the cumulative effects of ongoing suppression attempts (i.e., rebound) should not be as great.

Predictions based on age-related increases in readily available distraction are also relatively consistent with predictions following from older adults’ compensatory efforts and greater reliance on reactive control. Over time, younger adults would be expected to initially show declines in recurrence and subjective reactions to thought suppression as their strategic search for distractors improves with practice. However, because they must strategically search for distractors, as resources become depleted over repeated suppression attempts, recurrence as well as subsequent perceived difficulty will eventually rise. To the extent that distractors come to mind automatically for older adults, they should show less change in recurrence and difficulty across suppression attempts because reductions in controlled processing resources will not interfere greatly with access to the more automatically generated distractors.

To summarize, we expect older adults will show relatively more stable responses across the repeated thought suppression attempts, compared to younger adults who may experience initial practice-related declines in the frequency and duration of thought recurrence and in self-reported suppression difficulty, followed by a later uptick on these variables due to continual reliance on resource-demanding proactive control and less readily available distractor thoughts. This study reanalyzes Beadel et al., who used a modified thought suppression paradigm that required four separate suppression-then-monitor sequences to test for age differences in mean level and trajectory of change over time in frequency and duration of thought recurrence, as well as perceived difficulty among older and younger adults. To our knowledge, this is the first study to investigate age-related changes in reaction to *repeated* thought suppression attempts. Examining how effectively one can control or suppress a thought the first time it comes to mind is very different than understanding the effectiveness of thought suppression attempts when they are required over and over again. Given the repetitive nature of intrusive thinking [24], [25], [32], this approach is critical to understanding change in thought recurrence patterns in an ecologically valid way.

## Methods

All procedures were approved by the University of Virginia Institutional Review Board for Social and Behavioral Sciences. All participants provided their written informed consent to participate in this study. This consent process was approved by the University of Virginia Institutional Review Board for Social and Behavioral Sciences. While we do have three participants under the age of 18 in our sample, the psychology department participant pool at the University of Virginia, from which our sample was recruited, has a mandatory rule that any participant under age 18 must have a parent or guardian sign and return a written consent form on their behalf before they are able to begin participation in any study in the department. This consent procedure has been approved by the University of Virginia ethics committee (Institutional Review Board for the Social and Behavioral Sciences - University of Virginia).

### Participants

There were 42 younger adults (mean age = 18.9, SD = 1.5, range = 16–25) and 40 older adults (mean age = 76.0, SD = 7.9, range = 66–92) in the current study. The younger adult group was 57% female, and reported race as 69% Caucasian, 21% Asian, 5% African American, and 5% Other. The older adult group was 65% female, and reported race as 95% Caucasian, and 5% Other. Younger participants were recruited from a university department of psychology participant pool and older adults were recruited from the community via flyers and newspaper advertisements. Inclusion was based on age (18–30 years for the younger group, and 65 years or above for the older group). The exclusion criterion was the inability to meet a minimum score of 24 on the Mini-Mental Status Exam (MMSE), a measure of cognitive functioning. The lowest MMSE score in the overall sample was a 24.5 (range = 24.5–30), and younger (*M* = 29.15, *SD* = .94) and older adults (*M* = 28.70, *SD* = 1.40) did not significantly differ in performance, though there was a trend for older adults to score lower (*t*(80) = 1.74, *p* = .087, *d* = .39).

### Materials

#### Thought sequences

Participants underwent four thought suppression-then-monitor sequences, each of which included three periods. The first (practice focusing) period presented a thought and instructed participants to think about the thought as much as possible for 30 seconds. In the second period, they were asked to suppress the thought for 105 seconds. In the third period, they were asked to monitor their thoughts for 105 seconds. During the suppression period, participants were instructed: “Try not to think about the thought. If you do think about the thought, it’s very important that you hold down [the spacebar], but try your best not to think about the thought at all.” During the monitoring period, participants were instructed: “Think about whatever you would like. It could be the thought you thought about before, or it could be anything else. Hold down [the spacebar] any time you think about the given thought.” A Java-based computer program designed for the current study presented the thought stimulus, collected ratings about the thought, and recorded the frequency and duration of thought recurrence using the keyboard spacebar.

The four thoughts used for the thought sequences differed in valence (a positive thought: “I hope I win the lottery”, and a negative thought: “I hope my friend gets in a car accident”) and age-relevance (an older age-relevant thought: “I will lose my memory and forget my friends and family”, and a younger age-relevant thought: “I will never succeed in my career”; see [10]).

#### Reactions to thought suppression and thought monitoring

During each of the thinking periods, participants were asked to press the spacebar whenever they thought about the target thought, and to hold it down for however long the thought occurred, providing measures of both frequency and duration. They were to release the space bar when they thought about anything else. After each suppression and monitoring period, participants answered two questions. The first, used as a manipulation check to assess suppression effort, was, “Rate how hard you tried not to think about the thought during the last period.” Participants responded on a five-point Likert scale ranging from one, “I didn’t try at all” to five, “I tried as hard as possible.” The second, designed to assess perceived difficulty with suppression, was, “Rate how much difficulty you experienced trying to keep this thought out of your mind”. This item was rated on a five-point Likert scale ranging from one, “No difficulty” to five, “Extreme difficulty.”

#### Dementia screener

The Mini-Mental Status Exam (MMSE; [33]) was administered to participants to screen for symptoms of dementia by assessing orientation, attention, memory, language, and visuospatial processing. Per the recommendations of Tombaugh and McIntyre [34], a cut-off score of 24 was used as an exclusion criterion in the current study. No participants scored below 24.

### Procedure

Consent was obtained from all participants prior to the commencement of the study. Participants were told that they were going to be participating in a study examining the way people react to certain thoughts, some of which may be intrusive or undesirable. During informed consent, there was no mention of age or cognitive decline to reduce priming older adult concerns about age-related cognitive decline. Participants underwent the four thought suppression-then-monitor sequences, with randomized presentation of thought content. After each suppression and monitoring period, participants rated their level of suppression effort and perceived difficulty with suppression. Upon the conclusion of the thinking sequences, participants completed a demographics form. The experimenter then administered the MMSE to each participant. This test was placed at the end of the study so as not to prime participants’ concerns related to cognitive functioning during the study. Finally, participants were debriefed and compensated for their time. (Note that additional materials were included in this study, but are not reported here because they were not the focus of the present reanalysis. These materials were the Positive and Negative Affect Schedule-Negative Affect subscale (PANAS-NA [35]), the State-Trait Anxiety Inventory (STAI [36]), and the Subjective Units of Distress Scale (SUDS). The Trail-making subtests 2 and 4 from the Delis Kaplan Executive Function System (TM [37]) were also included in this study as an executive function measure.).

### Analytic Plan

Our primary questions concern whether older and younger adults differ systematically in their mean level and trajectories of change in thought recurrence (both frequency and duration), and their subjective difficulty during thought suppression and thought monitoring periods. Because we predicted that age differences would be more likely during thought suppression (when the aging-vulnerable, strategically controlled operating process is most active) than during monitoring, we analyzed data from suppression periods separately from that of monitoring periods. This yielded six sets of analyses, such that each of the three dependent variables–frequency, duration and difficulty–was examined within the thought suppression periods and within the thought monitoring periods.

To account for both within- and between-individual variation, we estimated multilevel models of change [39]. Specifically, level-1 components of these models estimate parameters for within-individual variation across the four thought sequences, and level-2 components account for between-individuals variation in initial levels and patterns of change trajectories (linear or curvilinear). The level-2 portions of the models allow inclusion of predictor variables, like age group, as explanations for between-individual variation. Random level-2 effects (unexplained interindividual variation) were estimated for the intercepts in all models, and for *TIME* and *TIME* ^2^ effects when these were included in Models B and C. However, given that our fundamental questions concern systematic effects of age group, we focus on the structural or *fixed effect* level-2 model estimates, that is, on whether the estimated average, or prototypical, initial level and patterns of change differ for the two age groups. For each dependent variable, the sequence of three nested models listed below was estimated to systematically examine the effect of age and how it may interact with linear and quadratic curvilinear change over repeated suppression attempts (note, given that little research has been done with more than two occasions of thought suppression measurements, we thought it prudent to systematically test for possible nonlinear patterns to the extent possible, the single-turn quadratic model was the estimation limit of our four occasions. Thus, we did not attempt to estimate cubic change trajectories because four waves of data are insufficient for that degree of modeling complexity [39]). In the models, “*AGE”* is a dummy-coded variable indexing the difference between older and younger adults (coded 1 and 0, respectively), *“TIME”* indexes the linear effect of the four thought sequences, and *TIME* ^2^ indexes a quadratic curvilinear effect.

Model A: Age only (provides a baseline main effect estimate of *AGE*, collapsed across the four thought sequences)

Model B: Age and time (introduces main effects of linear and curvilinear change, *TIME* and *TIME* ^2^, yielding average change trajectory estimates constrained to be equal for older and younger adults)

Model C: Age and time interactions (allows change trajectories to vary for older and younger adults by including *AGE*x*TIME* and *AGE*x*TIME* ^2^ product terms)

Our focus is on the parameter estimates for age, time and their interaction; not on overall model fit comparisons. As such, Model C provides the most comprehensive answer to our questions because the interactions test whether changes over time in thought suppression and monitoring behaviors vary with age. Nonetheless, results for the simpler Models A and B are provided in Table 1 so that parameter estimates can be seen in all cases, and described below in those instances when Model C parameter estimates are not significant. Given that variability due to thought content differences might have inhibited our ability to observe age and time effects, we controlled for possible main effects of thought content by including three orthogonal contrast codes as covariates in all models. These orthogonal codes indexed the following thought content contrasts: (1) Lottery thought vs. all others; (2) Accident thought vs. memory and career thoughts; and (3) Memory vs. career thought.

**Table 1:** Fixed Effect Estimates and Deviance Statistics from the 3-Model Sequence for Each Dependent Variable within Each Thought Condition.

			Model A		Model B			Model C		
			Age only		Age and Time			Age and Time Interactions		
Frequency (log) *Suppression*										
*AGE* initial status	*Younger*	γ_00_	1.31	***	1.42	***		1.48	***	
	*Older (difference)*	γ_01_	−0.26	*p* = .08	−0.28	*p* = .05		−0.40	*	
Rate of change	*TIME*	γ_10_			−0.19	*		−0.29	*	
	*TIME^2^*	γ_20_			0.05	*p* = .06		0.08	*	
	*AGE* * *TIME*	γ_11_						0.22		
	*AGE* * *TIME^2^*	γ_21_						−0.05		
**Goodness-of-fit**						*LRT*			*LRT*	
	Deviance (−2*LL*)		577.6		563.6	14/7		561.1	2.5/2	
										
Frequency (log) *Monitor*			**Model A**		**Model B**			**Model C**		
*AGE* initial status	*Younger*	γ_00_	0.94	***	0.95	***		0.98	***	
	*Older (difference)*	γ_01_	−0.15		−0.16			−0.23		
Rate of change	*TIME*	γ_10_			0.07			−0.04		
	*TIME^2^*	γ_20_			−0.03			0.01		
	*AGE* * *TIME*	γ_11_						0.22		
	*AGE* * *TIME^2^*	γ_21_						−0.07		
**Goodness-of-fit**						*LRT*			*LRT*	
	Deviance (−2*LL*)		552.8		545.3	7.5/7		542.8	2.5/2	
Duration (ms log) *Suppression*			**Model A**		**Model B**			**Model C**		
*AGE* initial status	*Younger*	γ_00_	8.07	***	8.34	***		8.53	***	
	*Older (difference)*	γ_01_	−0.57	*p* = .07	−0.65	*		−1.03	*	
Rate of change	*TIME*	γ_10_			−0.27			−0.80	*	
	*TIME^2^*	γ_20_			0.05			0.21	*	
	*AGE* * *TIME*	γ_11_						1.12	*	
	*AGE* * *TIME^2^*	γ_21_						−0.35	*	
**Goodness-of-fit**						*LRT*			*LRT*	
	Deviance (−2*LL*)		1224.6		1218.7	5.9/7		1213	5.7/2	
Duration (ms log) *Monitor*			**Model A**		**Model B**			**Model C**		
*AGE* initial status	*Younger*	γ_00_	7.49	***	7.62	***		7.77	***	
	*Older (difference)*	γ_01_	−0.65	*p* = .06	−0.65	*p* = .06		−0.95	*	
Rate of change	*TIME*	γ_10_			0.14			−0.22		
	*TIME^2^*	γ_20_			−0.10			0.01		
	*AGE* * *TIME*	γ_11_						0.75		
	*AGE* * *TIME^2^*	γ_21_						−0.23		
**Goodness-of-fit**						*LRT*			*LRT*	
	Deviance (−2*LL*)		1245.2		1237.4	7.8/7		1234.8	2.6/2	
Difficulty (1–5 rating) *Suppression*			**Model A**		**Model B**			**Model C**		
*AGE* initial status	*Younger*	γ_00_	2.45	***	2.71	***		2.94	***	
	*Older (difference)*	γ_01_	−0.27		−0.31			−0.80	***	
Rate of change	*TIME*	γ_10_			−0.29			−0.71	**	
	*TIME^2^*	γ_20_			0.06			0.16	*	
	*AGE* * *TIME*	γ_11_						0.89	**	
	*AGE* * *TIME^2^*	γ_21_						−0.23	*	
**Goodness-of-fit**						*LRT*			*LRT*	
	Deviance (−2*LL*)		915.5		896.8	18.7/7**		887	9.8/2**	
Difficulty (1–5 rating) *Monitor*			**Model A**		**Model B**			**Model C**		
*AGE* initial status	*Younger*	γ_00_	1.79	***	1.79	***		1.82	***	
	*Older (difference)*	γ_01_	0.18		0.14			0.06		
Rate of change	*TIME*	γ_10_			0.17			0.00		
	*TIME^2^*	γ_20_			−0.07			−0.01		
	*AGE* * *TIME*	γ_11_						0.35		
	*AGE* * *TIME^2^*	γ_21_						−0.11		
**Goodness-of-fit**						*LRT*			*LRT*	
	Deviance (−2*LL*)		833.7		826.9	6.8/7		825.2	1.7/2	

*Note*.

* p<.05;

** p<.01;

*** p<.001.

All models include thought-content contrast codes as covariates. *AGE* is coded 0 for younger adults (n = 42) and 1 for older adults (n = 38). *TIME* (coded 0–3) is the linear effect of the four successive thought sequences of a given instructional condition (Suppression or Monitor) and *TIME^2^* is the quadratic effect. Frequency and Duration are log-transformed to reduce positive skew, with respective transformed ranges of 0 to 2.9 and 3.8 to 11.6 (un-transformed ranges are 1–17 for Frequency and 46–104,890 ms for Duration). Difficulty ranges from 1 to 5.

The *AGE initial status: Younger* parameter is the model-estimated value during the first thought sequence for the younger group (the model intercept because this group is coded 0). The *AGE initial status: Older (difference)* parameter is the older group's difference from this intercept value. In Models A and B, the *AGE initial status: Older (difference)* parameter can be interpreted as a level effect of age. In Model C, however, by including the age-by-time interaction terms, the *AGE initial status: Older (difference)* parameter now reflects the estimated older adult difference during the first thought suppression sequence. Analogously, in Model B, the *TIME* and *TIME^2^* parameters index the linear and quadratic change trajectories for the full sample, collapsed across age. In Model C, however, the *TIME* and *TIME^2^* parameters now index the linear and quadratic change trajectories for younger adults (the group coded 0), and the interaction terms, *AGExTIME* and *AGExTIME^2^*, estimate how the older adults’ trajectories differ from those of the younger adults.

Deviance (−*2LL*) = −2*the sample log-likelihood, an index of the difference between the current model and a perfectly fitting saturated model (Singer & Willett, 2003). It follows a standard chi square distribution. *LRT* (Likelihood Ratio Test) = ?−*2LL*/?*df* from the previous model. In addition to the fixed effects shown here, all models also included estimates of the within- and between-person variance and covariance parameters that are characteristic of multilevel models. This is why, for example, the change in *df* from Model A to B is 7 rather than 2.

Results of random level-2 effect estimates (Intercept, *TIME* and *TIME* ^2^) are not shown. Intercepts varied significantly across individuals in all models, but effects of *TIME* and *TIME* ^2^ did not vary significantly for five of the six dependent variable-thought instruction conditions. The exception was for perceived difficulty in the suppression condition, where significant variation was evident for both the *TIME* and *TIME* ^2^ effects. That is, after accounting for the systematic effect of age, significant interindividual variability remained in participants’ patterns of perceived difficulty over time.

To minimize positive skew, the thought frequency and duration variables were log-transformed for all analyses. Raw means and standard deviations for each dependent variable are listed in Table 2 separately for the suppression and monitoring periods, by age group and thought suppression sequence. Alpha was set at.05 for evaluating whether model parameter estimates were non-zero. *TIME* is coded sequentially 0–3, so that intercept estimates can be interpreted as the initial value of a given dependent variable at the first thought sequence. Following Singer and Willett’s [39] multilevel modeling notation, unstandardized regression parameter estimates are denoted with the Greek letter gamma as follows: *γ*_01_ for the coefficient of the effect of *AGE*, *γ*_10_ for *TIME,* and *γ*_20_ for *TIME* ^2^.

**Table 2:** Mean and SD for Dependent Variables by Thought Condition and Age Group for Each of the Four Successive Thought Sequences.

	Suppression				Monitor			
	Younger		Older		Younger		Older	
	Mean	SD	Mean	SD	Mean	SD	Mean	SD
Frequency (space bar presses)								
TS1	**5.5**	3.1	**4.0**	3.1	**3.3**	2.4	**2.8**	2.5
TS2	**4.7**	3.3	**3.7**	3.7	**3.4**	2.6	**2.9**	2.6
TS3	**4.3**	3.1	**4.2**	3.8	**3.2**	2.2	**3.2**	2.0
TS4	**4.5**	3.0	**3.7**	3.0	**3.5**	3.2	**2.6**	2.2
Duration (seconds)								
TS1	**12.5**	18.4	**9.6**	23.0	**11.7**	20.2	**7.5**	23.3
TS2	**9.3**	11.9	**8.5**	19.0	**5.9**	8.1	**7.6**	23.3
TS3	**5.7**	6.5	**8.4**	17.2	**9.7**	19.1	**3.7**	5.1
TS4	**9.1**	10.3	**3.9**	5.8	**7.3**	11.6	**4.2**	16.4
Difficulty (rating 1–5)								
TS1	**2.9**	1.1	**2.1**	1.1	**1.8**	0.8	**1.9**	1.0
TS2	**2.4**	1.0	**2.2**	1.2	**1.9**	1.0	**2.0**	1.2
TS3	**2.2**	1.1	**2.3**	1.2	**1.8**	1.0	**2.2**	1.0
TS4	**2.3**	1.0	**2.1**	1.0	**1.7**	1.0	**1.8**	0.8

*Note*. TS1-4 index the four successive thought sequences within respective suppression and monitor conditions.

For the sake of brevity, only the fixed effect parameter estimates (the averages) are listed for each predictor. The random effects, variances and covariances of the predictors in the models were also estimated in all models, but are not listed in the Table 1 since they are not focal in our analyses. Complete model estimates are available from the second author on request.

## Results

Without exception, the thought-suppression periods yielded higher scores than the thought-monitoring periods on the frequency and duration of thought recurrence, and ratings of perceived difficulty across each of the four thought sequences within both age groups (see Table 2).

### Thought Suppression Effort: Manipulation Check

Participant reports of suppression effort were compared across the thought-suppression and thought-monitoring thinking periods to serve as a manipulation check. As expected, participants reported expending more effort suppressing during thought-suppression than during thought-monitoring, *t*(79) = 7.84, *p*<.001, *d* = .88, further supporting the validity of the manipulation.

### Age Differences in Mean Level of Thought Suppression Reactions

#### Frequency

**Figure 1:**
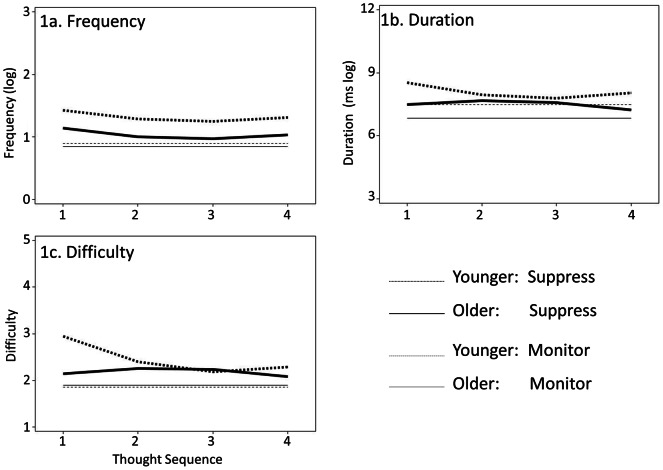
Model-estimated trajectories for thought frequency, duration, and perceived suppression difficulty across the four thought sequence occasions by age group. Monitor plots in 1a and 1c reflect the grand mean (age differences *ns*).

During the suppression periods, the Model C estimates of the interaction of age with linear and quadratic time were non-significant, so we interpreted the Model B age effect estimate as a constant effect across the four sequences. Specifically, older adults indicated less frequent thought recurrence than did younger adults across thought sequences, *γ*_01_ = −.28, *p* = .05 (see Figure 1a). In the monitor periods, none of the models yielded significant effects of age or time, so the most parsimonious estimate of the level effect of age was the non-significant one from Model A, *γ*_01_ = −.15, *p* = .26.

#### Duration

During suppression, because the significant age-by-time interaction estimates of Model C indicated different change trajectories for the two age groups, our estimate of age difference in level only applied to the first thought sequence. Older adults reported shorter durations at this initial measurement, γ_01_ = −1.03, p = .01. During the monitor periods, since neither age-by-time interaction estimates nor time estimates were significant (Models C and B), we interpreted the trend for a level effect of age in Model A. Older adults trended toward shorter average durations during monitoring than younger adults across thought sequences, γ _01_ = −.65, p = .06, suggesting an age effect that was similar in direction but smaller in magnitude than the significant effect observed in the suppression periods. See Figure 1b.

#### Difficulty

Once again, there was a significant age difference in mean level during suppression. As seen in Model C of Table 1 and graphically in Figure 1c, older adults had lower estimated average difficulty ratings at the first thought sequence, γ_01_ = −.80, p = .001. In contrast, the age groups did not significantly differ from one another in mean level during monitoring periods, γ_01_ = .18, p = .21 (see Model A of Table 1).

Summary results of the six sets of models are listed in Table 1. Statistically significant (or trending; i.e., p = .05 or.06) age differences in level were found for four of the six sets of models, always in the direction that older adults had lower or smaller thought suppression reactions than younger adults. Age differences were observed during suppression for all three dependent variables (frequency, duration, and perceived difficulty), but only one age difference was evident during the monitoring periods (a trend effect for duration). This pattern supports the prediction that age differences would be primarily observed during suppression when the aging-vulnerable operating process is thought to be most active.

### Age Differences in Trajectory across Repeated Suppression Attempts

For each analysis, we began by interpreting the parameter estimates of Model C, because these included the age by time interaction estimates of central interest in our study. When these interaction effects were non-significant, we interpreted the main effect parameter estimates for time in Model B and for age in either Model B or A, depending on whether effects of time were significant.

#### Frequency

During the suppression periods, results for Model C indicated that the change trajectory did not vary significantly with age; i.e., estimates for *AGE*x*TIME* and *AGE*x*TIME* ^2^ parameters were non-significant (*γ*_11_ = .22, *p* = .21; *γ*_22_ = −.05, *p* = .32). Thus, Model B provided the most parsimonious estimates of the effects of time; namely, a negative linear effect (*γ*_10_ = −.19, *p* = .03) was evident in the initial negative slope for the lines, and a positive quadratic effect (*γ*_20_ = .05, *p* = .06) was evident in the leveling, then up-ticking, from the second to the fourth thought sequence (see Figure 1a). In the monitor periods, the Model C estimates for *AGE*x*TIME* and *AGE*x*TIME* ^2^ parameters again were non-significant (*γ*_11_ = .22, *p* = .13; *γ*_21_ = −.07, *p* = .11), and the main effects of time were also non-significant in Model B (*TIME*, *γ*_10_ = .07, *p* = .34; *TIME* ^2^, *γ*_20_ = −.03, *p* = .21). Thus, across the monitor periods, the grand mean for frequency was the best approximation for both age groups (represented in the figure by the flat lines, which reflect the lack of a significant effect of time).

#### Duration

During the suppression periods, different age-linked patterns of linear and quadratic change (*AGE*x*TIME*, *γ*_11_ = 1.12, *p* = .02, and *AGE*x*TIME* ^2^, *γ*_22_ = −.35, *p* = .02) were observed in Model C. See Figure 1b. These significant estimates of interaction effects tell us that the age groups differed from one another in their trajectories, but not whether either group’s trajectory differed from zero. To test whether the parameter estimates of change were significantly non-zero during suppression for either age group, separate follow-up models for each age group were estimated. These analyses indicated that the linear and quadratic change effects were significantly non-zero for the younger adults (*TIME*, *γ*_10_ = −.77, *p* = .01; *TIME* ^2^, *γ*_20_ = .20, *p* = .03), but not for the older adults (*TIME*, *γ*_10_ = .27, *p* = .46; *TIME* ^2^, *γ*_20_ = −.12, *p* = .32). These within-age-group estimates suggest that the change pattern observed in Figure 1b for younger adults – initial decrease, then leveling and up-ticking – reflected a significant departure from a flat trajectory both in linearity and curvature, but this was not true for the older adults’ pattern of relative stability, with linear and quadratic change estimates that did not reliably differ from zero. During the monitor periods, stability was evidenced by both age groups, with no reliable main or interaction effects of time estimated in either Models B or C (Model B time estimates*: γ*_10_ = .14, *p* = .56; *TIME* ^2^, *γ*_20_ = −.10, *p* = .20; Model C *AGE*x*TIME* and *AGE*x*TIME* ^2^ estimates: *γ*_11_ = .75, *p* = .12; *γ*_21_ = −.23, *p* = .12).

#### Difficulty

During suppression periods, Model C estimates indicated different linear (γ_11_ = .89, *p* = .01) and quadratic (γ_21_ = −.23, *p* = .04) change trajectories for the age groups. Follow-up analyses within each age group revealed that change over time was again significantly non-zero for the younger adults, but not for the older adults. Specifically, both the linear and quadratic change estimates for difficulty were significant for the younger adults (γ_10_ = −.68, *p* = .002; γ_20_ = .15, *p* = .026), but neither was significant for the older adults (γ_10_ = .18, *p* = .50; γ_20_ = −.07, *p* = .42). Thus, the younger adults’ perceptions of difficulty controlling intrusive thoughts were initially greater than older adults’, but then dropped over the next two thought sequences before plateauing and up-ticking slightly, whereas the older adults changed little across the four thought sequences. During monitoring, there were no significant main effects of time (Model B, *γ*_10_ = .17, *p* = .23; *γ*_20_ = −.07, *p* = .14) or age-by-time interaction effects (Model C, *γ*_11_ = .35, *p* = .23; *γ*_21_ = −.11, *p* = .19), so, again, the same constant level was plotted for both age groups in Figure 1c.

Summary results of the six sets of models are again listed in Table 1. During suppression, both older and younger adults exhibited significant linear and quadratic patterns of change in frequency across time. For both groups, frequency initially decreased, then leveled before up-ticking slightly at the end. This same pattern was evidenced by young adults for duration and difficulty. However, older adults’ non-significant estimates for both linear and quadratic change patterns for duration and difficulty indicate stability across repeated suppression attempts. Monitoring periods revealed no significant estimates of time, age, or interactions between time and age across all three models.

#### Impact of thought type

As a check that our estimates of age effects were not artifacts of controlling for thought-type, all models were also estimated without thought-type covariates. The same essential pattern of age effects held, with only two instances of lost statistical precision for age-effect estimates. Specifically, for recurrence frequency in the suppression condition, the estimated main effect of *AGE* in Model B changed from γ_01_ = −.28, *p* = .05, to γ_01_ = −.27, *p* = .06, without thought-content covariates, while for perceived difficulty, the estimate for the interaction of *AGE* with quadratic change (Model C under suppression) went from γ_22_ = −.23, *p* = .04, to γ_22_ = −.19, *p* = .10.

### Supplementary Analysis: Moderation by Executive Functioning

A “contrast score” (i.e., difference score) of the Trail-making subtests 2 and 4 was included as a moderator in an exploratory analysis to evaluate whether level of executive functioning deficits would moderate the age effects, but no significant interactions with age were found. This null result is not surprising given that the Trail-Making Test is a neuropsychological measure designed to detect executive function deficits in cognitive set shifting [38], an executive function likely less relevant to thought suppression than others, such as sustained attention or goal maintenance.

## Discussion

The present study used multilevel modeling to examine age differences in the frequency and duration of thought recurrence, as well as subjective reports of suppression difficulty, across repeated thought suppression sequences. As predicted, age differences in level and trajectories of change were more apparent during suppression periods than monitoring periods. During suppression, younger adults’ initial levels of recurrence (both frequency and duration), and subjective ratings of thought suppression difficulty were higher than those of older adults, but then gradually dropped, likely due to practice. On all variables, younger adults’ reactions began to rise again on the last measurement occasion, possibly due to suppression-induced resource depletion. In contrast, for all outcomes, older adults’ responses remained relatively stable across time.

### Suppression Periods versus Monitor Periods

The finding that age differences in both level and trajectory were primarily observed during suppression periods is consistent with major theoretical accounts of age-related cognitive change [7], which hypothesize that strategically controlled processes are more likely than automatic processes to decline with age. Further, the operating process, assumed to be especially active during suppression periods, and thought to involve effortful, consciously controlled processing, has been linked to prefrontal activity [40], [41]. Because the frontal lobes are particularly susceptible to age-related changes [42], age differences should be more apparent during suppression periods than monitoring periods within the thought suppression paradigm.

### Initial Level Age Differences in Reaction to Thought Suppression

Older adults tended to have shorter and less frequent thought recurrences, and reported suppression periods to be less difficult than younger adults, especially during the first thought sequence. Previous research using the White Bear Suppression Inventory [43] to measure age differences in everyday thought suppression tendencies produced similar findings [44], albeit relying on retrospective self-report. The present experimental findings strengthen the validity of this effect and draw upon theories of age-related cognitive change as a means by which to understand this age difference. The findings did not support level predictions of the Compensation account, which hypothesized an absence of age differences in recurrence and *greater* older adult perceived difficulty, and the possibility that older adults would increase on all three variables across time if compensatory resources were depleted. The Dual-Mechanisms of Control and Distraction accounts were more consistent with the observed results, suggesting that age differences in control strategies and/or age-related sensitivity to distraction may be developmentally important to understanding how reactions to thought suppression change as we age. In the present context, reactive control and susceptibility to distraction may have made it easier for older adults to conserve resources during suppression, reducing the need for compensation.

It is interesting to compare our account of reactive control in the context of thought suppression to the aging and emotion regulation literature. This literature often argues that older adults tend to use antecedent (i.e., proactive) emotion regulation strategies to avoid situations that could lead to negative affect (e.g., [45], [46]), with the idea that employing the extra effort to prevent negative affect will be worthwhile in the long run because managing the affect once activated can be costly. Our results suggest that older adults’ choice of proactive versus reactive modes of control may be highly context-dependent, and vary as a function of what may ultimately best conserve resources.

Older adults’ shorter, less frequent recurrence during suppression, as observed in the present analyses, is consistent with the trend of greater younger adult recurrence (frequency and duration combined) reported by Beadel et al. [10], though the effects were stronger in this reanalysis. The present analytic approach refines our understanding of this trend, suggesting that it was primarily driven by higher younger adult recurrence early on in the sequence. While Magee and Teachman [9] observed no age differences in recurrence, it is possible that age differences were present early-on, but were subsequently obscured by increasing similarity in recurrence between younger and older adults as time went on. Given that the thought suppression and monitoring periods were longer in Magee and Teachman than those used in the current analyses, and that only the average recurrence was examined across these periods in Magee and Teachman, this seems plausible. Neither Beadel et al. nor Magee and Teachman observed main effects of age in perceived difficulty during suppression that would correspond to the level differences we found using multilevel modeling (though Magee and Teachman [9] did find that older adults reported more difficulty than younger adults during monitoring). Here again, it is possible that age differences were present early-on but later obscured by increasing similarity between the groups over time. These contrasting results point to the advantage of modeling change over repeated attempts or time more carefully, rather than simply collapsing across time when assessing a resource-demanding cognitive process.

### Time Trajectory Age Differences in Reaction to Thought Suppression

Our findings for all but one of the dependent variables (duration during monitoring) indicated a quadratic average trajectory for younger adults across time, such that they started higher than older adults, habituated with practice and then increased on the final thought sequence. In contrast, older adults showed relative stability across time and across variables compared to younger adults, patterns consistent with both the Dual-Mechanisms of Control account and the Distraction account.

For younger adults, these patterns of change suggest that they are attempting to use thought suppression and that they are initially getting better at it, based on the steep early-sequence declines in their thought suppression reactions. During this period where they seem to benefit from practice, younger adults may be quickly expending cognitive resources and, in accordance with the Dual-Mechanisms of Control account, employing resource demanding proactive control while doing so. This appears to work well up until the last measurement occasion when their reactions to thought suppression begin to rise, perhaps because, as time goes on, younger adults’ controlled processing resources may dwindle. Older adults’ patterns of stability suggest that age-related changes in controlled processing may result in relative stability in measures of thought recurrence, at least in the short term. Their vulnerability to distraction may reduce the need for a resource demanding search for distractions from the forbidden thought, thus sparing their cognitive resources. In addition, older adults may be relying on more reactive modes of control that would be less resource depleting. Together, distractibility and reactive control may ultimately allow older adults to expend controlled processing resources relatively slowly and steadily across measurement occasions, thus leading to stability in thought suppression reactions across time. It is possible, however, that even longer thinking periods and sustained suppression attempts may tax older adults’ processing resources to the point that reactive control and distraction are no longer sufficient to prevent an eventual increase in thought recurrence and related suppression outcomes.

### Limitations and Future Directions

These findings should be considered in light of several limitations. First, the present study used an extreme groups design with respect to age. While this approach is common within the aging literature and acceptable if age-related cognitive change is linear across the lifespan, as is the case for many cognitive variables [47], it also carries the potential to obscure non-linear cognitive change during middle age. Further, our use of a very young adult college student sample may have somewhat limited our opportunity to observe age differences in thought suppression. Accumulating evidence suggests that the frontal cortex and its corresponding controlled processing abilities are not fully developed until around the middle of the second decade of life [48], [49]. Thus, it is possible that a slightly older, young adult sample would have provided a more distinct comparison group to our older adult sample, given these young adults would have presumably had a more fully developed frontal cortex. Notwithstanding, extreme age group comparisons are common in the aging literature. Many studies employing these designs have used young adult samples with age means under 25 years [50]–[52], including studies of executive functioning differences that are linked to thought suppression abilities. Notably, these studies guided our predictions in the present paper [26], [24]. Thus, while future research replicating these results with an older young-adult sample or, better yet, a cross-sectional lifespan sample, represents a logical next step, we do believe that much can be learned from the current comparison.

Second, resource depletion was not directly measured. While research in the resource-depletion literature strongly suggests that suppressing unwanted information over similar durations to ours is depleting [13]–[15], future research looking at dual-task performance costs, suppression under cognitive load, or post-suppression performance costs on a controlled processing measure (such as Stroop color naming) could more definitively speak to the involvement of resource depletion in the observed data patterns. If age differences in controlled processing resources are, in part, driving the patterns observed in the present analyses, then young adults who are asked to suppress following a cognitive load, should produce a pattern of thought recurrence more similar to that of older adults without a cognitive load. The inclusion of physiological indices that may be related to thought suppression reactions, such as changes in blood glucose (indicative of resource depletion [13]) or blood pressure (indicative of difficulty and effort [53]) could add valuable convergent evidence. As an alternative to directly manipulating cognitive load or measuring changes in blood glucose, future research could also begin to better address the role of age-related cognitive change in thought suppression reactions by including individual differences measures in executive function measures, such as sustained attention or goal maintenance as moderating variables.

Third, while the present paper considers three plausible theoretical explanations (Compensation, Dual-Mechanisms of Control, and Distraction) for age differences in thought suppression performance, alternative explanations do exist. Age differences in processing speed, alertness, response criterion, strategy use, and initial thought activation may have also contributed to the observed patterns. For example, age differences in the time course of semantic activation have been reported such that older adults show slightly slower activation [54], [55]. Further, there is accumulating evidence in the memory literature that some inhibitory processes may be spared during aging. For example, comparable rates of forgetting have been observed across a variety of different memory paradigms, suggesting little evidence for an inhibitory deficit on these tasks [56]. It is possible that older adults simply do not experience inhibitory deficits in the context of the current study paradigm. Future research controlling for or manipulating these factors will help to further refine our understanding of how age-related changes in cognition impact thought suppression. Age-related changes in socioemotional functioning, such as a relative tendency for older adults to preferentially process positive information [57], may also have interesting influences on thought suppression at different ages. For example, examining thought valence as a moderating variable would be an interesting direction for future research.

Finally, in order to reduce the number of study conditions, given the complexity of the multiple thought sequence design, Beadel et al. [9] had participants complete a thought suppression period followed by a thought monitoring period for each sequence, as opposed to using a typical thought suppression design that randomizes participants to suppression versus monitoring instructions at the beginning of the study. Because this design lacked randomization of initial suppression and monitoring instructions, we cannot examine how initial suppression vs. monitoring may have differentially led to rebound effects.

### Conclusions

Despite these limitations, the present study advances our understanding of relationships between thought suppression and cognitive processing in younger and older adults. Our models were most consistent with the Dual-Mechanisms of Control and Distraction accounts, but neither account provided a perfect fit. More likely, a combination of these two accounts contributed to the general age patterns observed across our models. If reactive control strategies and distractibility reduce the amount of controlled processing resources older adults must expend during thought suppression, then older adults may have sufficient controlled processing resources to accomplish the demands of the thought suppression task, at least for the period assessed in the present study. The present analyses support the hypothesis that frontally-mediated, age-related changes in controlled processing impact thought recurrence and thought suppression difficulty. However, they suggest that, at least in the context of thought suppression, age-related declines in frontal function may not be all bad. Rather, they may reduce the resource depleting nature of thought suppression attempts, in turn leading to lower levels of thought recurrence and more stable responses to suppression over time. While this interpretation may seem to contradict decades of research in cognitive aging that have focused on age-related declines, it is consistent with a growing body of evidence that suggests not all age-related change harms performance [58], and in some cases age related change can improve performance [57]. For instance, old age seems to enhance emotional stability and preferential processing of positively valenced information [28], [29], [57]. Consistent with this idea, changes in cognitive processing may support stability in older adults’ responses to thought suppression, thereby providing some protection from the negative consequences that repeated thought suppression attempts can elicit.
